# Validity of the German Version of the FCR-1r Questionnaire in Patients with Urologic Cancer

**DOI:** 10.1159/ort/aecag002

**Published:** 2026-08-03

**Authors:** Can Dümdüz, Sebastian Sage, Sofia Kugler, Günter Niegisch, Christian Schulz-Quach, Ralf Schäfer, André Karger, Ulrike Dinger, Dominik Fugmann

**Affiliations:** a Clinical Institute of Psychosomatic Medicine and Psychotherapy, Medical Faculty and University Hospital Düsseldorf, Heinrich-Heine-University Düsseldorf, Düsseldorf, Germany; b Center for Integrated Oncology Aachen, Bonn, Cologne and Düsseldorf (CIO ABCD), Düsseldorf, Germany; c Department of Psychosomatic Medicine and Psychotherapy, LVR Hospital Düsseldorf, Heinrich Heine-University Düsseldorf, Düsseldorf, Germany; d Department of Urology, Medical Faculty and University Hospital Düsseldorf, Heinrich-Heine University Düsseldorf, Düsseldorf, Germany; e Division of Psychosocial Oncology, Department of Supportive Care, University Health Network – Princess Margaret Cancer Centre, Toronto, ON, Canada; f Wilson Centre, University of Toronto, Toronto, ON, Canada; g Department of Psychiatry, Temerty Faculty of Medicine, University of Toronto, Toronto, ON, Canada

**Keywords:** Psychooncology, Surveys and questionnaires, Fear of cancer recurrence, Urologic neoplasm, Psychometrics

## Abstract

**Introduction:**

Fear of cancer recurrence (FCR) is a prevalent and distressing issue for a significant proportion of cancer patients across various disciplines, yet it remains one of the most frequently unaddressed psychosocial needs. A key barrier to identifying affected individuals is the lack of brief screening tools suitable for clinical settings. The objective of this research is to validate the one-item FCR screening tool (revised Fear of Cancer Recurrence-1 [FCR-1r]) in a German cohort of urologic cancer patients. Validating such an ultra-brief measure may help address this clinically relevant gap and serve as a viable, clinically applicable alternative to lengthy questionnaires.

**Methods:**

Statistical analyses were conducted using R software. Criterion validity was assessed against the Concerns About Recurrence Questionnaire-4 (CARQ-4). Convergent validity was tested against the Fear of Progression Questionnaire – Short Form (FoP-Q-SF) and Generalised Anxiety Disorder-7 (GAD-7). Discriminant validity was tested against the Health Literacy Survey-European-Questionnaire 16 (HLS-EU-Q16). Validity was assessed using chi-square tests with phi coefficients, accuracy measures, negative and positive predictive values, sensitivity, specificity and odds ratios.

**Results:**

The final analysis sample consisted of 236 participants with complete data on all study measures. The FCR-1r demonstrated excellent criterion validity with respect to the CARQ-4 (χ^2^[1] = 101.97, *p* < 0.001, φ = 0.66), with an overall accuracy of 84% (95% CI: 79–89%). Sensitivity was 84%, and specificity was 85%. The analysis of convergent validity revealed a moderate association with both the FoP-Q-SF (χ^2^[1] = 20.25, *p* < 0.001, φ = 0.29; accuracy 62% [95% CI: 56–68%]) and the GAD-7 (χ^2^[1] = 11.82, *p* < 0.001, φ = 0.22; accuracy 64% [95% CI: 58–70%]). The analysis of discriminant validity showed no significant association between the FCR-1r and health literacy (HLS-EU-Q16): χ^2^(1) = 3.44, *p* = 0.064, φ = 0.12.

**Conclusion:**

The German version of the FCR-1r demonstrates good psychometric properties, confirming its validity as a tool for assessing FCR in clinical practice.

## Introduction

The number of cancer diagnoses and cancer-related deaths are on the rise [1]. Forecasts regarding the further development of the epidemiology of cancer diseases indicate a potential worsening of this issue in upcoming years [1]. The International Agency for Research on Cancer estimates that approximately one in five people will develop cancer during their lifetime [1]. Based on demographic projections, the authors anticipate a 77% rise in new cancer diagnoses by 2050, compared to 2022 figures [1]. This trend is further compounded by the growing number of cancer survivors, partly attributable to advances in medical care [2–4].

With this life-changing diagnosis [5], cancer patients often suffer from comorbidities as well as psychosocial distress [6–8]. The most frequent diagnoses include depressive disorders, adjustment disorders, and anxiety disorders [6–8]. Among other relevant forms of psychosocial distress, fear of cancer recurrence (FCR) emerges as particularly significant. FCR represents an adaptive anxiety response to the threat of disease progression or recurrence that may promote treatment compliance and tertiary prevention. However, at pathological levels, FCR can substantially impair quality of life, potentially enduring for decades following cure [9]. A recent comprehensive meta-analysis confirmed a significant association between elevated FCR and symptoms of depression, anxiety, and general emotional distress [10]. Furthermore, a qualitative systematic review and meta-synthesis of patients’ experiences revealed that FCR can manifest with trauma-like qualities, including intrusive re-experiencing, avoidance behaviour, and heightened arousal in response to cancer-related triggers or memories [11].

In medical literature, multiple references can be found that consistently show that a significant part of cancer patients – from different medical disciplines – suffer from FCR [12–16]. It affects 31% of testicular cancer survivors [12] and 35% of head and neck cancer patients [13], 47% of women with newly diagnosed gynaecological cancer [14] and 70% of breast cancer survivors [15]. Through a systematic review of multiple research findings, Simard et al. [16] concluded that an average of 49% of cancer survivors report moderate to high levels of FCR. Thus, it is evident that FCR represents a relevant comorbidity among cancer patients and, due to both improved survival rates [2–4] and the anticipated increase in new cancer diagnoses [1], is likely to gain even greater significance over time.

Nevertheless, FCR manifests as one of the most common unfulfilled psychosocial needs of cancer patients [17, 18]. This unmet need persists across both active treatment and post-treatment phases [18]. A significant challenge in establishing diagnoses to enable subsequent treatment lies in the questionnaires used for this purpose [19]: A systematic review of various multi-item questionnaires for FCR revealed that while several longer assessment instruments (>10 items) with sufficient reliability and validity data exist [20], these comprehensive measures – though valuable in academic research – are often impractical for clinical settings due to their length [19].

The aim of this research is to validate a one-item screening questionnaire for FCR (revised Fear of Cancer Recurrence-1 [FCR-1r]) in German for patients with urologic cancer. The validation of such a short screening instrument for FCR could potentially fill the gap previously mentioned and serve as an alternative (to long questionnaires), which might be more applicable in clinical settings.

## Methods

### Study Design and Participants

This study constitutes a secondary, cross-sectional analysis of data from a double-blind, randomised controlled trial. The protocol of the primary randomised controlled trial is registered at ClinicalTrials.gov (NCT06545188). For the present validation analysis, we used data from patients with urological cancer who were recruited during their inpatient treatment at the Department of Urology, University Hospital Düsseldorf, and who completed the relevant questionnaires during their inpatient stay. Data were collected using paper-based questionnaires.

The following inclusion criteria were applied:

- urological cancer (ICD-10: C60, C61, C62, C63, C64, C65, C66, C67, C68);
- age of at least 18 years;
- German language skills;
- sufficient cognitive ability; and
- capacity to consent.

No exclusion criteria were applied.

Between September 2024 and October 2025, the study personnel approached a total of 636 patients. Prior to seeking consent, 61 patients were found not to meet the predefined inclusion criteria: insufficient German language skills (*n* = 48), insufficient cognitive ability (*n* = 8), being under 18 years of age (*n* = 2), and having already participated (*n* = 3). Of the 575 eligible patients, 309 patients agreed to enrol in this study. After excluding 73 participants due to incomplete questionnaire data, the final analysis sample comprised 236 patients.

Among the 266 patients who declined to participate, the primary reasons were lack of interest (43.6%), perceived excessive burden (37.2%), lack of time (9%), and other factors (10.2%). The participant recruitment and inclusion process is illustrated in Figure 1.

**Fig. 1.**
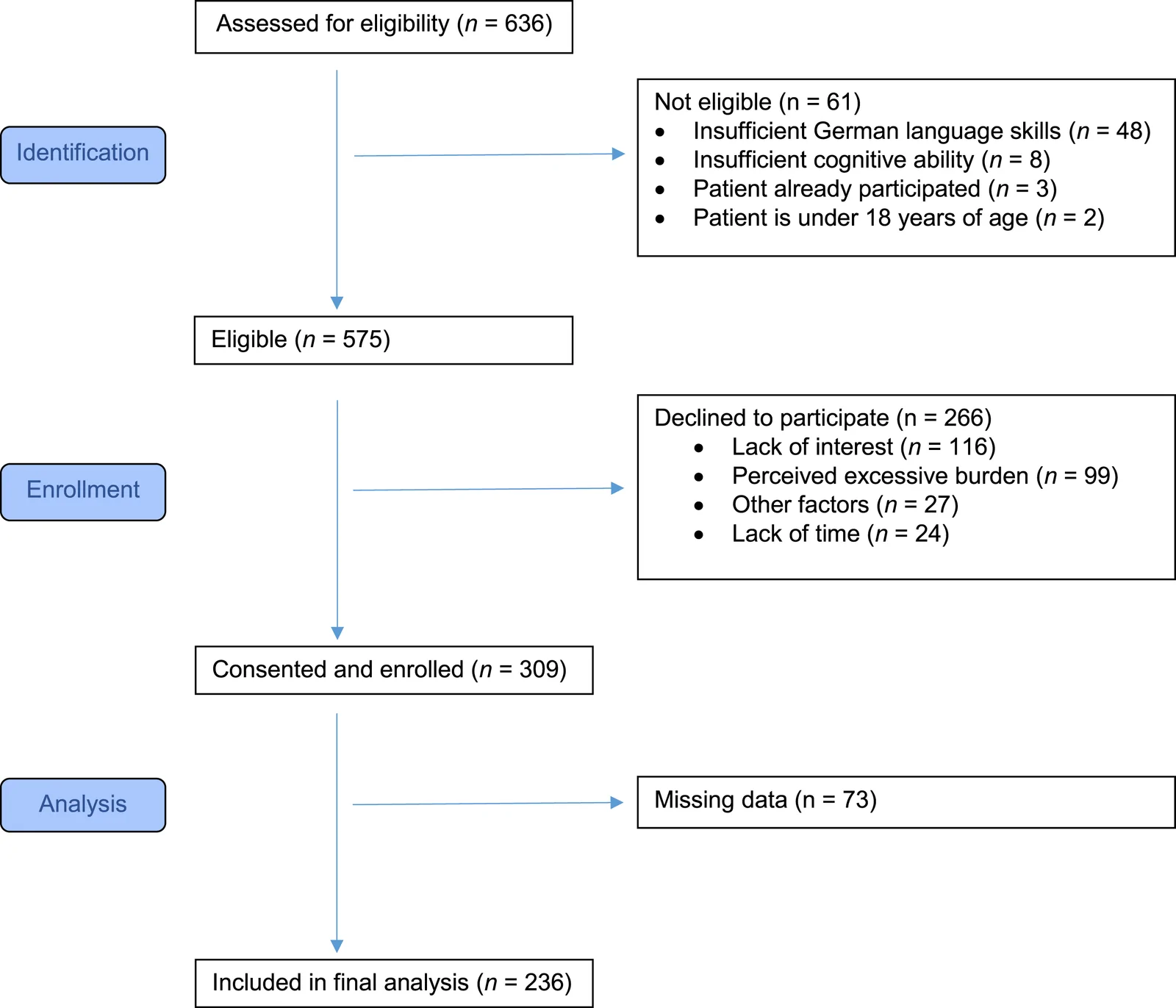
Flow diagram of participants at each stage of the study. Of 636 patients approached, 61 did not meet inclusion criteria. Of the 575 eligible patients, 309 agreed to participate. After excluding 73 participants due to incomplete questionnaire data, the final analysis sample consisted of 236 patients.

### Instruments

The following instruments were used to assess the criteria of interest:

- **FCR-1r:** The FCR-1r questionnaire is a single-item screening measure for FCR [21]. Patients are asked to rate their FCR on a scale from 0 to 10. In its primary validation study, a cut-off score of ≥5 was established to indicate clinically significant FCR. This cut-off was derived in a combined Australian sample (*n* = 107) from two studies: a cross-sectional study of patients with various cancer types (*n* = 54) and a single-arm feasibility study of patients diagnosed with breast, gynaecological, or head and neck cancer (*n* = 53). The single-item screener was translated using independent forward and back translation, reviewed by an interdisciplinary expert group, and examined by post hoc cognitive debriefing. The English and German versions of the FCR-1r are provided in online supplementary File 1 (for all online suppl. material, see https://doi.org/10.1159/ort/aecag002).
- **Concerns About Recurrence Questionnaire-4:** The Concerns About Recurrence Questionnaire-4 (CARQ-4) is a four-item screening measure for FCR [22]. This questionnaire consists of four items assessing FCR. For three of the items, responses are given on a scale from 0 to 10, whereas the fourth item requires participants to indicate a percentage from 0% to 100%. The latter is converted to a 0–10 scale for scoring purposes, resulting in a possible total score ranging from 0 to 40. A total score of ≥12 indicates clinically relevant levels of FCR. As shown by Hinz et al. [23], the Fear of Progression Questionnaire – Short Form (FoP-Q-SF) and the CARQ-4 demonstrate substantial overlap (*r* = 0.72), indicating a shared core construct, while differences in item composition suggest a differential weighting, with the FoP-Q-SF emphasising affective distress and the CARQ-4 additionally capturing a cognitive appraisal of recurrence probability.
- **FoP-Q-SF:** The German version of the 12-item FoP-Q-SF was administered [24]. It is based on the original 43-item Fear of Progression Questionnaire, which comprises five subscales, with the largest subscale being “Affective Reactions” (13 items) [25]. All items of the FoP-Q-SF are rated on a 5-point Likert scale ranging from “never” to “very often.” The total sum score ranges from 12 to 60, with a cut-off score of ≥34 indicating clinically relevant FCR.
- **Generalised Anxiety Disorder-7:** The Generalised Anxiety Disorder-7 (GAD-7) screener consists of 7 items, each rated on a four-point Likert scale [26]. Response options are “not at all,” “several days,” “more than half the days,” and “nearly every day,” which are scored as 0, 1, 2 and 3, respectively. Accordingly, the GAD-7 score ranges from 0 to 21. The cut-off score indicating elevated anxiety is 10 points.
- **Health Literacy Survey-European-Questionnaire 16:** The Health Literacy Survey-European-Questionnaire 16 (HLS-EU-Q16) is the short version of the original HLS-EU-Q47 [27]. It comprises 16 items that assess four dimensions of health literacy: accessing, understanding, evaluating and applying health information. All items are rated on a 4-point Likert scale ranging from “very easy” to “very difficult.” Responses are dichotomised (1 = “very easy”/“fairly easy”; 0 = “fairly difficult”/“very difficult”) and summed to a total score. Based on this score, health literacy is categorised as adequate (13–16 points), problematic (9–12 points) or inadequate (1–8 points). For statistical analysis, a binary variable was created using a cut-off score of ≥13 points to indicate adequate health literacy, while scores below 13 were classified as not sufficient, including both problematic and inadequate levels.

### Measures for Sample Characteristics

Sociodemographic and clinical characteristics of the sample were assessed using a study-specific screening form and a modified version of the psycho-oncological basic documentation [28]. The screening form provided information on age, tumour diagnosis, and level of education. The modified psycho-oncological basic documentation included information on gender, relationship status, parental status, employment situation, metastatic disease, date of initial cancer diagnosis, current disease status, treatments in the past 2 months, other relevant somatic comorbidities, use of psychopharmacological drugs or opioids, and history of psychological or psychiatric treatment. These measures were used exclusively for the descriptive characterisation of the study sample and were not included in the psychometric validation analyses. Variables derived from these instruments were summarised in Table 1 (sample demographic and clinical characteristics).

**Table 1.** Sample demographic and clinical characteristics (*n* = 236)

Category	Value^a^
Age, years	
Range	20–91
Mean (SD)	62.6 (15.6)
Gender, *n* (%)	
Male	214 (90.7)
Female	22 (9.3)
Committed relationship^b^, *n* (%)	
Yes	194 (82.2)
No	41 (17.4)
Children^b^, *n* (%)	
Yes	167 (70.8)
No	68 (28.8)
Level of education^c^, *n* (%)	
>10 years	120 (50.8)
10 years	50 (21.2)
<10 years	61 (25.8)
Employment status^d^, *n* (%)	
Employed	73 (30.9)
On sick leave	36 (15.3)
Homemaker	5 (2.1)
Retired	135 (57.2)
Unemployed	6 (2.5)
Other	7 (3.0)
Cancer diagnosis (ICD-10), *n* (%)	
Prostate cancer (C61)	96 (40.7)
Bladder cancer (C67)	59 (25.0)
Testicular cancer (C62)	37 (15.7)
Renal cancer (C64)	29 (12.3)
Penile cancer (C60)	7 (3.0)
Other urological cancer (C63, C65, C66, C68)	8 (3.4)
Metastatic disease^e^, *n* (%)	
Yes	73 (30.9)
No	117 (49.6)
Unknown	42 (17.8)
Time since initial diagnosis, months^f^	
Range	0–266
Mean (SD)	27.6 (49.4)
Current disease status, *n* (%)	
First diagnosis	156 (66.1)
Second primary cancer	30 (12.7)
Recurrence	33 (14.0)
Remission	2 (0.8)
Unknown	15 (6.4)
Treatment within the last 2 months^d^, *n* (%)	
Surgery	142 (60.2)
Radiotherapy	9 (3.8)
Chemotherapy	45 (19.1)
Other systemic therapy	6 (2.5)
Cancer-targeted medication (e.g., immunotherapy)	26 (11.0)
Hormone therapy	13 (5.5)
None	52 (22.0)
Other	9 (3.8)
Other relevant somatic comorbidities, *n* (%)	
Yes	93 (39.4)
No	140 (59.3)
Not known	3 (1.3)
Current medication: Psychopharmacological drugs/Opioids, *n* (%)	
Yes	18 (7.6)
No	210 (89.0)
Not known	8 (3.4)
History of psychological/psychiatric treatment (including current), *n* (%)	
Yes	33 (14)
No	198 (83.9)
Not known	5 (2.1)

^a^ Percentages may not sum to 100 due to rounding.

^b^ Data were available for 235 participants; 1 value was missing.

^c^ Data were available for 231 participants; 5 values were missing.

^d^ Participants could select multiple applicable options; therefore, percentages do not sum to 100%.

^e^ Data were available for 232 participants; 4 values were missing.

^f^ Data were available for 225 participants; 11 values were missing.

### Statistical Analysis

Statistical analyses were conducted using R software. The following procedures were applied:

- **Criterion validity:** CARQ-4.
- **Convergent validity:** FoP-Q-SF and GAD-7.
- **Discriminant validity:** HLS-EU-Q16.

Criterion validity of the FCR-1r was examined using a chi-square test with Phi coefficients as a measure of effect size. In addition, diagnostic performance was evaluated by computing sensitivity, specificity, positive and negative predictive values, overall accuracy with 95% confidence intervals (CIs), and odds ratios. Criterion validity was examined using the CARQ-4 as the primary reference standard, as it most closely reflects the cognitive core construct of FCR assessed by the FCR-1r. The optimal cut-off score for the German FCR-1r was determined by conducting a receiver operating characteristic (ROC) analysis against the CARQ-4, maximising the Youden Index (shown in Fig. 2).

**Fig. 2.**
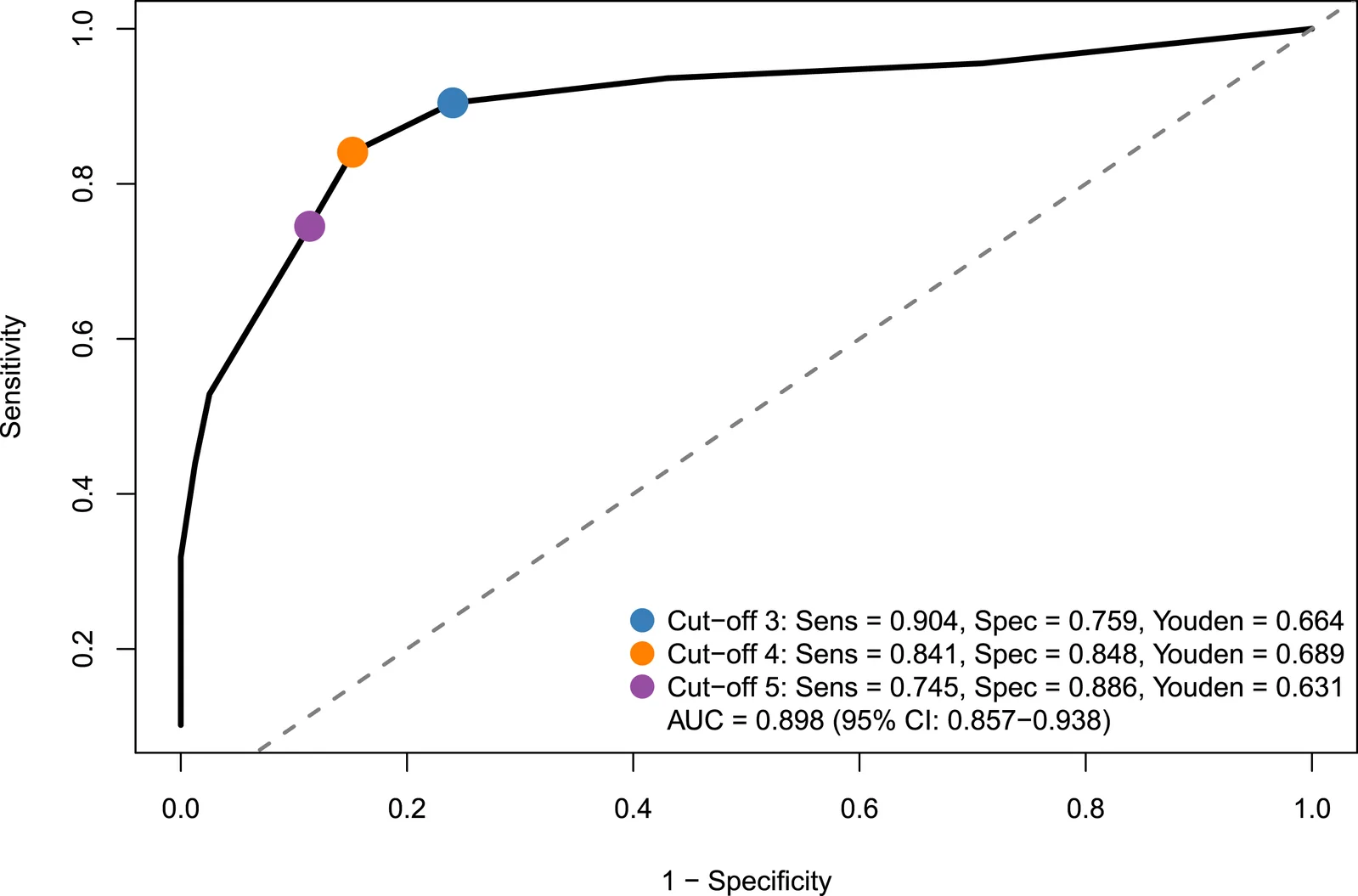
Receiver operating characteristics (ROC) curve of the FCR-1r questionnaire using CARQ-4 as reference standard. The area under the curve (AUC) was 0.898 (95% CI: 0.857–0.938). The optimal cut-off value determined by the Youden Index was 4, yielding a sensitivity of 84.1% and specificity of 84.8%. Additional cut-offs at 3 and 5 were evaluated to illustrate the trade-off between sensitivity and specificity at different threshold levels.

Convergent validity of the FCR-1r was examined using chi-square tests with Phi coefficients. Additional measures included sensitivity, specificity, positive and negative predictive values, overall accuracy with 95% CIs, and odds ratios. The FoP-Q-SF (assessing the related construct of fear of progression) and the GAD-7 (assessing general anxiety) served as external criteria.

Discriminant validity of the FCR-1r was examined using chi-square tests with Phi coefficients. Additional measures included sensitivity, specificity, positive and negative predictive values, overall accuracy with 95% CIs, and odds ratios. The HLS-EU-Q16 served as an external criterion for health literacy.

### Assessment of Psychometric Properties

The present study focuses on the validation of the FCR-1r. Reliability, particularly test-retest reliability, was not assessed, as this would require repeated measurements over time in a stable population, which is beyond the scope of this cross-sectional design.

## Results

### Sample Characteristics

The final analysis sample consisted of 236 participants with complete data on all study measures. The sample had a mean age of 62.6 years (SD = 15.6), ranging from 20 to 91 years, indicating a broad age distribution representative of the oncological population. The study included 214 males (90.7%) and 22 females (9.3%). The distribution of cancer types was as follows: prostate cancer (40.7%), bladder cancer (25.0%), testicular cancer (15.7%), renal cancer (12.3%), penile cancer (3.0%), and other urological cancers (3.4%). The mean time since diagnosis was 27.6 months (SD = 49.4) among the 225 patients with available data on initial diagnosis (11 missing values).

To assess the potential for attrition bias resulting from the exclusion of participants with incomplete questionnaire data, baseline characteristics of completers (*n* = 236) and non-completers (*n* = 73) were compared. Available baseline characteristics suggested no major differences between completers and non-completers and are presented in online supplementary File 2.

Using the established cut-off scores, a substantial proportion of the sample reported clinically significant levels of FCR. Based on the CARQ-4, 66.5% (*n* = 157) of participants were identified as having clinically relevant FCR. The prevalence was similar when assessed with the optimised FCR-1r (cut-off: ≥4), which identified 61.0% (*n* = 144) of participants above the clinical threshold.

### Criterion Validity

To determine the optimal cut-off score for the German version of the FCR-1r in our sample, a ROC analysis was performed using the CARQ-4 as the criterion standard. The analysis yielded an area under the curve of 0.898 (95% CI: 0.86–0.94), indicating excellent diagnostic accuracy. The original proposed cut-off of ≥5 [21] demonstrated a sensitivity of 74.5% and a specificity of 88.6% against the CARQ-4 in our sample. To optimise the instrument for screening purposes in clinical practice, where high sensitivity is prioritised to minimise false negatives, the Youden Index was calculated for all possible cut-off points. The cut-off score of ≥4 yielded the maximum Youden Index (J = 0.689).

Consequently, the cut-off score of ≥4 was selected for all subsequent analyses. As this threshold was derived in the present sample, it should be considered preliminary pending external validation. Using this optimised cut-off, the FCR-1r demonstrated excellent criterion validity with respect to the CARQ-4 (χ^2^[1] = 101.97, *p* < 0.001, φ = 0.66), with an overall accuracy of 84% (95% CI: 79–89%). Sensitivity was 84%, and specificity was 85%. The positive predictive value was 92%, and the negative predictive value was 73%. An odds ratio of 29.5 (95% CI: 13.95–62.32) indicates a strong association between the FCR-1r and the CARQ-4. Detailed diagnostic performance metrics for the evaluated FCR-1r cut-off scores are summarised in Table 2.

**Table 2.** Sensitivity and specificity of FCR-1r cut-off scores using the CARQ-4 (≥12) as criteria for clinical FCR

Cut-off	Sensitivity, %	Specificity, %	Youden Index	PPV, %	NPV, %
≥0	100	0	0	66.5	–
≥1	95.5	29.1	0.247	72.8	76.7
≥2	93.6	57.0	0.506	81.2	81.8
≥3	90.4	75.9	0.664	88.2	80.0
≥4^a^	84.1	84.8	0.689	91.7	72.8
≥5	74.5	88.6	0.631	92.9	63.6
≥6	52.9	97.5	0.503	97.6	51.0
≥7	43.9	98.7	0.427	98.6	47.0
≥8	31.8	100	0.318	100	42.5
≥9	15.9	100	0.159	100	37.4
≥10	10.2	100	0.102	100	35.9

FCR-1r, revised Fear of Cancer Recurrence-1; CARQ-4, Concerns About Recurrence Questionnaire-4; PPV, positive predictive value; NPV, negative predictive value.

^a^ Optimal FCR‐1r cut‐off for FCR screening.

### Discriminant Validity

The analysis of discriminant validity showed no significant association between the FCR-1r and health literacy (HLS-EU-Q16): χ^2^(1) = 3.44, *p* = 0.064, φ = 0.12. Diagnostic performance indicated an accuracy of 45% (95% CI: 39–52%), with a sensitivity of 56% and a specificity of 32%. The positive predictive value was 52%, and the negative predictive value was 35%. The odds ratio of 0.6 (95% CI: 0.34–0.99) suggests a weak negative association.

### Convergent Validity

The analysis of convergent validity revealed a moderate association between the FCR-1r and the FoP-Q-SF (χ^2^[1] = 20.25, *p* < 0.001, φ = 0.29). Diagnostic performance showed an accuracy of 62% (95% CI: 56–68%), with a sensitivity of 79%, specificity of 51%, a positive predictive value of 52%, and a negative predictive value of 78%. The odds ratio was 3.9 (95% CI: 2.16–7.08).

Similarly, a moderate association was found with the GAD-7 (χ^2^[1] = 11.82, *p* < 0.001, φ = 0.22). Diagnostic performance indicated an accuracy of 64% (95% CI: 58–70%), with a sensitivity of 70%, specificity of 54%, a positive predictive value of 73%, and a negative predictive value of 50%. The odds ratio was 2.7 (95% CI: 1.55–4.66). Associations between the FCR-1r and the external validation measures are summarised in Table 3.

**Table 3.** Correlations of the FCR-1r with validation measures

Type of alidity	Measure	Chi-square test	Phi φ	Accuracy (95% CI)	Sensitivity, %	Specificity, %	PPV	NPV	OR (95% CI)
Criterion validity	CARQ-4	χ^2^(1) = 101.97, *p* < 0.001	0.66	84 (79–89)	84	85	92	73	29.5 (13.95–62.32)
Convergent validity	FoP-Q-SF	χ^2^(1) = 20.25, *p* < 0.001	0.29	62 (56–68)	79	51	52	78	3.9 (2.16–7.08)
Convergent validity	GAD-7	χ^2^(1) = 11.82, *p* < 0.001	0.22	64 (58–70)	70	54	73	50	2.7 (1.55–4.66)
Discriminant validity	HLS-EU-Q16	χ^2^(1) = 3.44, *p* = 0.064	0.12	45 (39–52)	56	32	52	35	0.6 (0.34–0.99)

FCR-1r, Fear of Cancer Recurrence-1 revised; CARQ-4, Concerns About Recurrence Questionnaire-4; FoP-Q-SF, Fear of Progression Questionnaire – Short Form; GAD-7, Generalised Anxiety Disorder-7; HLS-EU-Q16, Health Literacy Survey-European-Questionnaire 16; CI, confidence interval; PPV, positive predictive value; NPV, negative predictive value; OR, odds ratio.

## Discussion

Our analyses demonstrate that the German version of the FCR-1r exhibits good criterion validity, as evidenced by strong agreement with the CARQ-4. The instrument also shows good convergent validity through its significant association with the FoP-Q-SF as well as the GAD-7, while the absence of a statistically significant association with health literacy suggests acceptable discriminant validity, although effect sizes indicate limited separation. These findings collectively support the FCR-1r as a psychometrically sound single-item solution for assessing FCR.

The current results align with international validation studies, such as those by Smith et al. [21], who reported similar psychometric properties for the English version. When contextualised within the landscape of existing FCR instruments, our findings highlight the unique advantage of an ultra-brief single-item format compared to more lengthy multi-item questionnaires, while maintaining comparable validity.

The brief administration time of <1 min makes the FCR-1r particularly suitable for routine clinical practice. Its implementation may facilitate the early identification of patients experiencing clinically relevant FCR, thereby enabling timely referral to psychosocial support. The findings further suggest that the instrument may be applicable across a range of healthcare settings, including oncology clinics, survivorship care programmes, and primary care.

Although the original validation study proposed a cut-off value of ≥5, ROC analyses in the present sample indicate that a slightly lower threshold (≥4) may be more suitable for German-speaking inpatient urological cancer patients. Given the primary aim of the FCR-1r as a screening instrument, prioritising sensitivity over maximal specificity appears clinically justified, as it reduces the likelihood of missing patients with clinically relevant FCR. This approach is consistent with established screening principles in psycho-oncology, where false-negative results are considered more problematic than false positives. Cut-off optimisation was conducted using the CARQ-4 as the criterion measure, as it most closely reflects the cognitive core of FCR. In contrast, the FoP-Q-SF was conceptualised as a measure of convergent validity rather than a diagnostic criterion, reflecting its broader focus on affective responses to illness progression. The moderate association observed between the FCR-1r and the FoP-Q-SF therefore supports conceptual convergence rather than diagnostic equivalence.

Generalisability of the present findings is strongest for German-speaking inpatient urological cancer patients treated at a university hospital, given the predominance of male participants and prostate cancer diagnoses in our sample. Psychometric performance and optimal screening thresholds may vary across cancer entities, treatment phases, and healthcare settings, where the prevalence and phenomenology of FCR as well as follow-up procedures differ. In particular, outpatient survivorship settings and cancer populations with a higher proportion of female patients may yield different optimal cut-off values. As predictive values are inherently prevalence-dependent, the diagnostic performance observed in this study should be interpreted as setting- and population-specific. Because the cut-off was derived and its diagnostic performance evaluated in the same dataset, the proposed threshold may be affected by optimism bias. Therefore, the threshold of ≥4 should be considered preliminary and requires confirmation in independent validation samples before being applied across cancer entities or care settings.

Several limitations should be considered when interpreting our results. The predominantly male sample (91%) from urological oncology limits the generalisability of findings to female cancer populations and other cancer types. Furthermore, as a single-item instrument, the FCR-1r does not allow for the assessment of internal consistency reliability, which is a common constraint of such ultra-brief measures. The cross-sectional design also precludes examination of test-retest reliability and predictive validity over time. Furthermore, the lack of cultural diversity in our sample warrants caution in applying these results to multicultural patient populations.

Approximately one quarter of enrolled participants were excluded because of incomplete questionnaire data. Comparison of available baseline characteristics showed broadly similar age, sex distribution and cancer diagnoses between completers and non-completers, suggesting that major selection bias is unlikely. Nevertheless, as only limited baseline information was available for excluded participants, the possibility of residual selection bias cannot be entirely excluded.

Future research should validate the FCR-1r in other cancer entities, particularly those with higher female representation, and in outpatient survivorship settings. Longitudinal studies are needed to establish the instrument’s predictive validity. In addition, implementation research may help to determine the feasibility and clinical impact of routine FCR screening using ultra-brief instruments in different healthcare contexts.

## Conclusion

The German FCR-1r demonstrates satisfactory psychometric properties as an ultra-brief screening instrument for FCR. Its single-item format provides a clinically feasible approach for routine identification of patients with elevated FCR in busy healthcare settings. By facilitating early detection, the FCR-1r may support timely referral to psycho-oncological care and contribute to addressing an important unmet need among German-speaking cancer populations.

## Statement of Ethics

This study was conducted in accordance with the principles of the Declaration of Helsinki. The study protocol was reviewed and approved by the Ethics Committee (EC) of the Medical Faculty of Heinrich Heine University Düsseldorf (approval no. 2024-2878). Written informed consent was obtained from each participant prior to participation in the study. To ensure the protection of research participants, all direct or indirect identifying information was excluded, and any potentially identifiable attributes were anonymised.

## Conflict of Interest Statement

The authors have no conflicts of interest to declare.

## Funding Sources

Financial support for this study was provided by the Gesellschaft von Freunden und Förderern der Heinrich-Heine-Universität Düsseldorf e.V. (GFFU) Heigl Foundation [82301]. The funder had no role in the design, data collection, data analysis, and reporting of this study.

## Author Contributions

D.F., C.D., S.S., and C.S.-Q. contributed to the study concept and design. Material preparation and data collection were carried out by D.F., C.D., and S.S. Analysis was performed by C.D., D.F., and R.S. The first draft of the manuscript was written by C.D. D.F., S.S., G.N., C.S.-Q., R.S., S.K., A.K., and U.D. commented on previous versions of the manuscript and read and approved the final manuscript.

## Data Availability

The data supporting the findings of this study are not publicly available due to their sensitive nature and because participants did not provide consent for public data sharing. However, the data are available from the corresponding author upon reasonable request; requests will be assessed in accordance with applicable data protection regulations.
